# Activation of endoplasmic reticulum stress and the extrinsic apoptotic pathway in human lung cancer cells by the new synthetic flavonoid, LZ-205

**DOI:** 10.18632/oncotarget.13535

**Published:** 2016-11-24

**Authors:** Yi Zhang, Xuefen Xu, Wei Li, Hanchi Miao, Shaoliang Huang, Yuxin Zhou, Yang Sun, Zhiyu Li, Qinglong Guo, Li Zhao

**Affiliations:** ^1^ State Key Laboratory of Natural Medicines, Jiangsu Key Laboratory of Carcinogenesis and Intervention, China Pharmaceutical University, Nanjing 210009, People's Republic of China; ^2^ School of Pharmacy, China Pharmaceutical University, Nanjing 210009, People's Republic of China

**Keywords:** LZ-205, apoptosis, ER stress, ROS, extrinsic apoptosis

## Abstract

It has been shown that flavonoids have anti-tumor activity. In this study, LZ-205, a newly synthesized flavonoid, was found to be effective in inducing apoptosis in human lung cancer cells *in vivo* and *in vitro*. Mechanistically, LZ-205 triggers reactive oxygen species (ROS)-induced endoplasmic reticulum (ER) stress and unfolded protein response, which could be reversed by silencing CHOP, a mediator of the ER stress-associated apoptosis. In addition, LZ-205-induced apoptosis is accompanied by the activation of both the mitochondrial apoptotic and extrinsic pathways, followed by decreased mitochondrial membrane potential (ΔΨm) and the alteration of the expression of mitochondria-related pro- and anti-apoptotic proteins. LZ-205 exhibits a potential antitumor effect in BALB/c nude mice bearing H460 tumor with low systemic toxicity. In summary, both the ROS-mediated ER stress pathway and the exogenous apoptotic pathway are involved in LZ-205-induced apoptosis *in vitro* and *in vivo*. Our data show a therapeutic potential of LZ-205 for the treatment of lung cancer.

## INTRODUCTION

Lung cancer is a major refractory disease which represents a serious threat to human health. Lung cancer is the number one cancer killer among both men and women in the world. Despite the recent findings on the molecular mechanism of lung cancer and the development of novel therapeutic drugs for its treatment, the 5-year survival rate for lung cancer patients has not changed substantially and remains dismal [1]. As the first-line treatment in lung cancer, chemotherapy helps at the expense of severe and debilitating side effects, but provides only modest survival benefit. Therefore, it is imperative to find novel targeted agents against lung cancer.

Apoptosis is well-known as a process of regulated cell death, which plays a major role not only in cell damage or stress, but also in normal development and morphogenesis [2]. This process results in DNA damage and is activated through two distinct pathways, namely the intrinsic or mitochondrial pathway and the extrinsic pathway [3].

The intrinsic pathway depends on various cell stress signals which usually lead to an imbalance of the Bcl-2 family of proteins in favor of apoptosis, mitochondria permeabilization with concomitant release of cytochrome c (Cyt-c) and eventual activation of caspase 9 [4]. Reactive oxygen species (ROS), such as superoxide (·O_2_¯), hydrogen peroxide (H_2_O_2_), and hydroxyl radicals (·OH), are mainly produced in the mitochondria and lead to oxidative damage [5]. It has been established that the endoplasmic reticulum (ER) is rather sensitive to oxidative stress damage and regulates the oxidative stress-induced cell death [6, 7]. Interfering with ER function, a condition termed “ER-stress”, leads to the accumulation of unfolded and misfolded proteins in the ER and activates the unfolded protein response (UPR), which can help cells adapt to harmful stimuli under physiological conditions. The UPR involves three well-established signaling receptors/proteins, namely the inositol-requiring protein-1α (IRE1α), the protein kinase RNA (PKR)-like ER kinase (PERK), and the activating transcription factor 6 (ATF6) [8]. These three receptors bind to the ER resident chaperone binding immunoglobulin protein (Bip/Grp78), which keeps them inactive. When too much misfolded or unfolded proteins accumulate, the ER releases the Bip protein from these complexes to assist with the folding of the accumulated proteins. The UPR reduces the accumulation of unfolded proteins and restores ER function to promote cell viability in adverse environments. However, when the stress condition remains unresolved, the UPR becomes a pro-apoptotic response [9].

Apoptosis is activated via the extrinsic or death receptor-mediated pathway when a specific ligand binds to its corresponding death receptor in the cell surface. Stimulation of death receptors, such as Fas and Trail, leads to the recruitment of the adaptor molecule Fas-associated death domain (FADD), which in turn recruits procaspase-8 to the receptor complex and eventually activates the apoptotic signaling cascade [10, 11].

**Figure 1:**
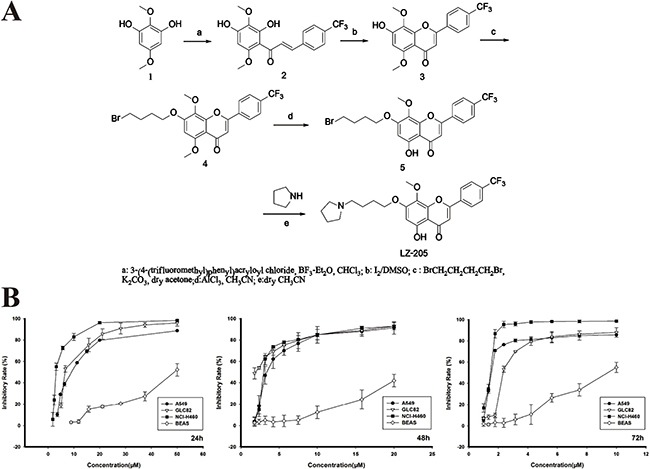
Growth inhibitory effect of LZ-205 *in vitro* **A**. The synthetic route of LZ-205. The molecular structure of LZ-205 (C_25_H_26_F_3_NO_5_, MW=477.47), Structure (1) is 2, 5-Dimethoxy-benzene-1, 3-diol (C_8_H_10_O_4_, MW=170.16). **B**. A549, GLC82, NCI-H460 and BEAS cells were treated with various concentration of LZ-205 for 24, 48 and 72 h. Cell viability was determined using MTT assay. Data were shown as Means ± SD (n = 3).

Natural flavonoids comprise a group of compounds widely found in plant sources. They have been extensively recognized to have anti-inflammatory, antioxidant, antiallergic, hepatoprotective, antithrombotic, and antiviral activities [12]. The most significant finding in flavonoids research in the last ten years is that flavonoids have anti-tumor activity [13]. Wogonin is one of the major compounds found in Scutellaria baicalensis and possesses potent anticancer activity. In order to improve its water solubility and druggability, LZ-205, 5-hydroxy-8-methoxy-07-(4-(pyrrolidin-1-yl)-butoxy)-2- (4-(trifluoromethyl)-phenyl)-4H-chromen-4-one), was synthesized. The synthetic route to LZ-205 is depicted in Figure 1A. Initially, the Friedel-Crafts acylation reaction between 2, 5-dimethoxy-benzene-1, 3-diol (1) and 3-(4-(trifluoromethyl) phenyl) acryloyl chloride yielded chalcone (2) which was then cyclized by iodine oxidation. The phenolic hydroxyl group at the C7 position of intermediate 3 was then alkylated using 1, 4-dibromobutane in acetone in the presence of K_2_CO_3_. After the selective demethylation, pyrrolidine groups were coupled with the halogenated hydrocarbons to produce LZ-205.

Considering the aforementioned factors, in this study we shed some light on the apoptosis-inducing effect of LZ-205 on human lung cancer cells and nude mice H460 xenograft. Our findings also revealed the molecular mechanisms whereby LZ-205 induces apoptosis *in vitro* and *in vivo*.

## RESULTS

### LZ-205 selectively inhibits human lung cancer cells but not normal cells

To evaluate cell viability *in vitro*, the MTT assay was used to determine the cell growth inhibitory effect of LZ-205 on human lung cancer cells. The IC_50_ values of A549, GLC82 and NCI-H460 cells at 24 h were 8.37 ± 0.17, 7.60 ± 0.74 and 3.93 ± 0.32 μM, respectively, as shown in Figure 1B. In addition, LZ-205 inhibited the growth of NCI-H460 cells in a time- and concentration- dependent manner, as indicated by the IC_50_ values measured at 48 and 72 h, which were 2.51 ± 0.18 and 1.31±0.09 μM, respectively. However, only a small percentage of cell death occurred in normal lung epithelial BEAS cells after treatment with the same concentration of LZ-205. These data suggested that LZ-205 had a selectively inhibitory effect on lung cancer cells but not on normal cells.

### LZ-205 induces apoptosis in human lung cancer cells

**Figure 2:**
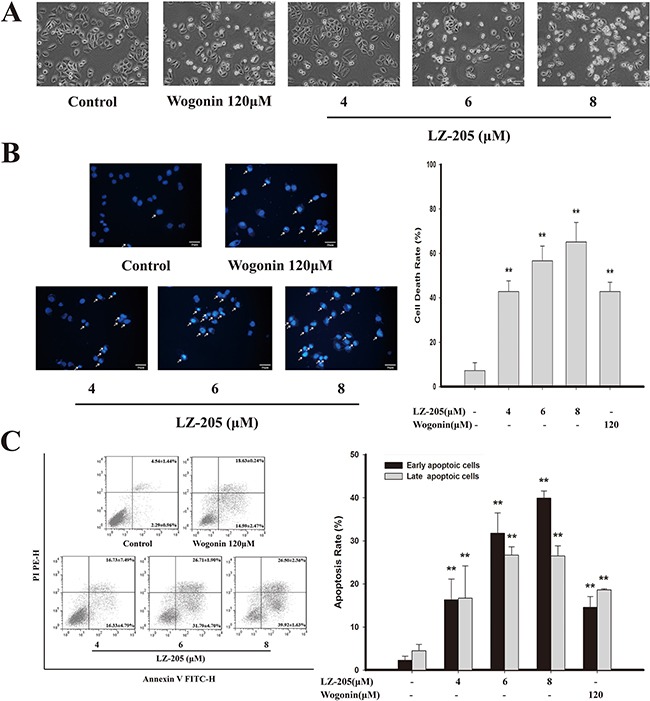
Apoptosis induced by LZ-205 in NCI-H460 cells Cells were treated with 4, 6 and 8μM LZ-205 for 24h. **A**. Observed under an inverted light microscope (200×). **B**. Stained with DAPI (400×). White arrow pointed to dead cells and cell death rate was analyzed. **C**. Annexin V/PI double-staining assay of NCI-H460 was analyzed by flow cytometry. Data were shown as Means ± SD for three independent experiments (*p<0.05 and ^*^*p<0.01 compared with Control).

Whether the anti-tumor activity of LZ-205 could be due to its apoptosis-inducing effect was investigated. We first examined the morphological changes in NCI-H460 cells treated with 4, 6, or 8 μM of LZ-205 or 120 μM of Wogonin for 24 h. Observation of the treated cells revealed that they were round and shrunken, indicating that they were apoptotic, whereas the untreated cells retained their normal size and shape, as shown in Figure 2A.

In order to confirm the occurrence of apoptosis, further morphological changes were evaluated by DAPI staining. The LZ-205-treated cells showed bright fluorescent staining, major characteristics of chromatin agglutination, nucleolus pyknosis, and apoptosis (white arrow), as shown in Figure 2B. With the increasing concentrations of LZ-205, more and more nuclear disintegration occurred along with DNA fragmentation.

LZ-205-induced apoptosis was further assessed by AnnexinV/PI staining (Figure 2C). A comparison with the control group (2.29±0.96%), revealed that the early apoptotic rate of LZ-205-treated (4, 6, and 8 μM) cells was increased to 16.33 ± 4.79%, 31.79 ± 4.70%, and 39.92 ± 1.63%, respectively. Additionally, the late apoptotic rate of cells treated with the same amounts of LZ-205 pointed toward the same trend. The early and late apoptotic rates of Wogonin-treated (120 μM) cells were 14.59±2.74% and 18.63±0.24%, respectively. In addition, the apoptosis inducing effects of LZ-205 were also found in other human lung cancer cells, such as A549 and GLC82, as shown in Supplementary Figure S1A.

Taken together, these results indicate that LZ-205 induces apoptosis in human lung cancer cells.

### The mitochondrial dependent pathway and exogenous apoptotic pathway are involved in LZ-205-induced apoptosis

**Figure 3:**
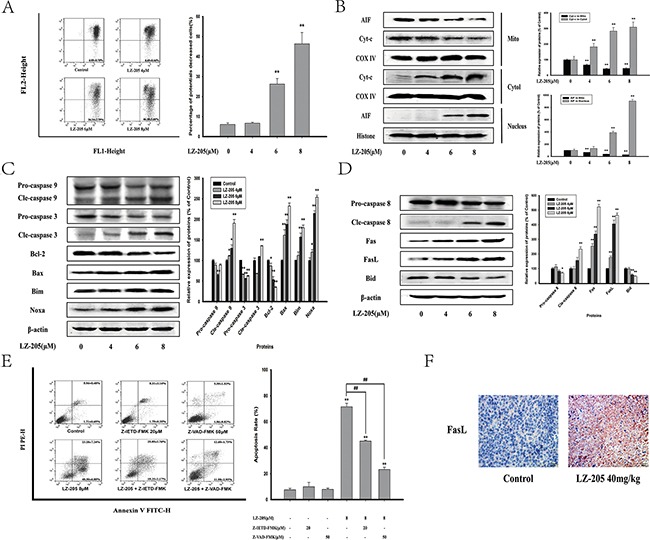
LZ-205 activates the mitochondrial apoptotic pathway and exogenous apoptotic pathway Cells were exposed to LZ-205 (4, 6 and 8 μM) for 24 h. **A**. Cells were stained with JC-1 and detected by flow cytometry. Then the percentage of ΔΨm collapsed cells was analyzed. **B**. Effect of LZ-205 on Cyt-c and AIF in NCI-H460 cells. Mitochondrial, cytosolic and nuclei fractions were subjected to western blot analysis. **C**. The protein of Caspase 9, Caspase 3, Bcl-2, Bax, Bim and Noxa were analyzed by western blotting. **D**. The protein of Caspase 8, Fas, FasL and BID were analyzed by western blotting. **E**. Cells were treated with/without 20 μM Z-IETF-FMK (a caspase 8 inhibitor), 50 μM Z-VAD-FMK (a pan caspase inhibitor) and 8 μM LZ-205 for 24 h. The apoptosis were analyzed with flow cytometry. **F**. Immunohistochemical detection of FasL protein level in mouse transplantation tumor tissues (100×). Results were means ± SD for at least three independent experiments (*p<0.05 and ^*^*p<0.01 compared with Control).

The change in mitochondrial membrane potential (MMP) change is a crucial stage in drug-induced apoptosis. The results of the MMP measurement showed a considerable decrease of the MMP level in LZ-205-treated cells (Figure 3A and Supplementary Figure S1B). The levels of Cyt-c were significantly increased in the cytosol of cells treated with LZ-205 for 24 h, whereas they were decreased in mitochondria (Figure 3B). Apoptosis inducing factor (AIF) in the LZ-205-treated group was also found to be decreased in mitochondria and increased in nucleus, which suggested that AIF was transferred to the nucleus from mitochondria. In addition, western blot analysis was conducted to further evaluate the effects of LZ-205-induced apoptosis on protein expression. The results of the analysis showed that cleaved-caspase 9, cleaved-caspase 3, Bim, Noxa, Bax were significantly upregulated, whereas the levels of pro-caspase 9, pro-caspase 3 and Bcl-2 were downregulated (Figure 3C). These results indicated that LZ-205 induces a mitochondrial dependent apoptotic pathway. At the same time, we found the cleaved-caspase 8, Fas and FasL levels were also increased, while expression of pro-caspase 8 and BID were decreased (Figure 3D), which indicated the activation of the extrinsic apoptotic pathway. To prove that LZ-205-induced apoptosis is caspase-dependent, we investigated the effect of Z-VAD-FMK, a pan caspase inhibitor. Additionally, Z-IETD-FMK, a caspase 8 inhibitor, was used to further verify the activating effect of LZ-205 on the extrinsic apoptotic pathway. The flow cytometry analysis showed that Z-IETD-FMK and Z-VAD-FMK suppressed LZ-205-induced apoptosis in NCI-H460 cells (71.58 ± 2.64% in the LZ-205 group vs. 45.10 ± 0.67% in the Z-IETD-FMK+LZ-205 group and 24.58 ± 1.28% in the Z-VAD-FMK+LZ-205 group), as shown in Figure 3E. These results suggested that LZ-205 induced both caspase 8 dependent and independent apoptotic pathway. Furthermore, immunohistochemistry (IHC) analysis of tissue samples revealed a significant up-regulation of FasL expression in the LZ-205-treated group and confirmed the occurrence of Fas-mediated apoptosis *in vivo* (Figure 3F).

Based on the above results, we concluded that LZ-205 induces both the intrinsic or mitochondrial apoptotic pathway and the extrinsic apoptotic pathway.

### LZ-205-induced ROS lead to apoptosis

**Figure 4:**
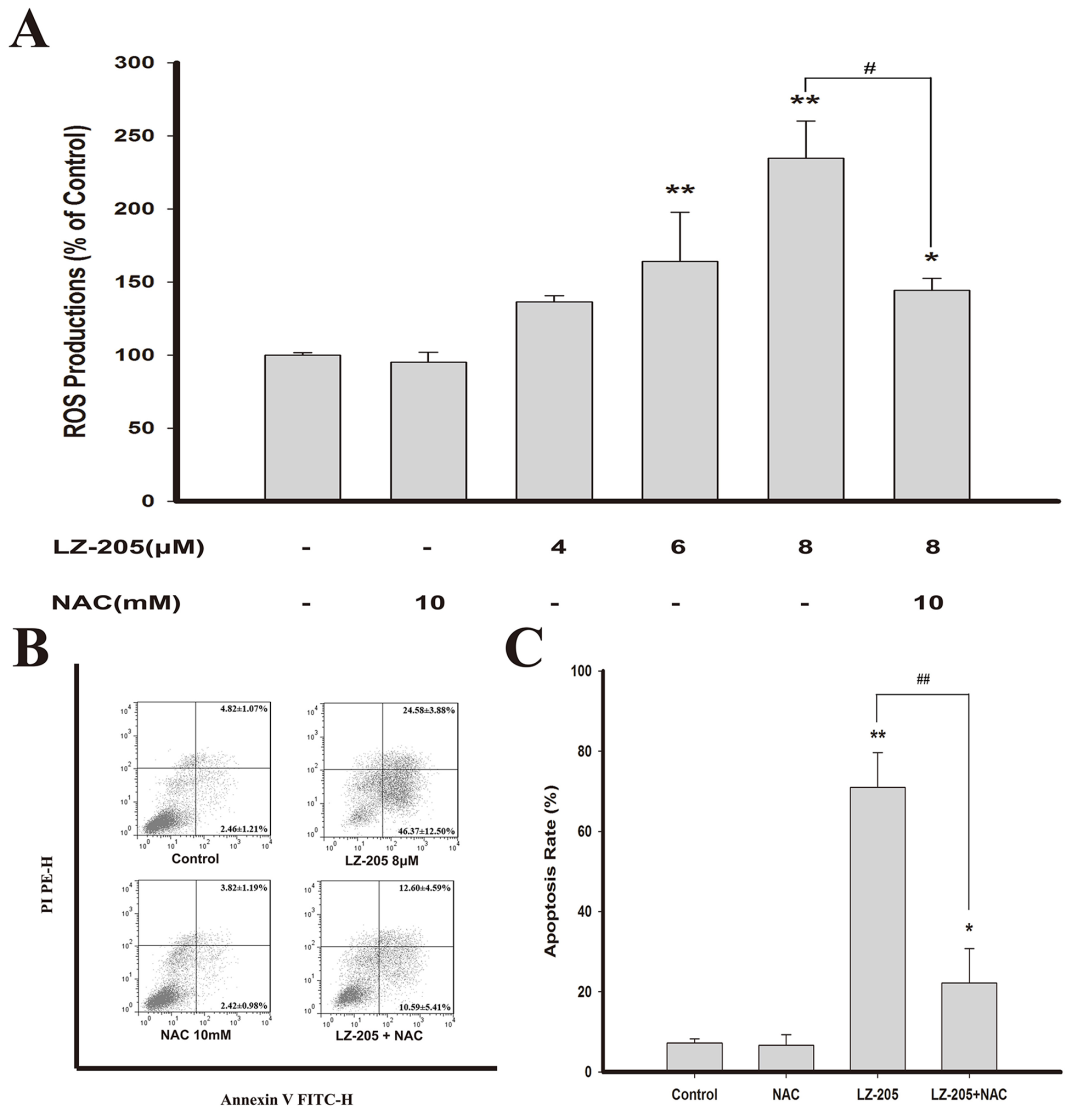
LZ-205 increased the level of ROS **A**. Cells were pretreated with/without 10 mM NAC for 1 h, and then exposed with/without LZ-205 for 12 h. Finally, the ROS level was detected by flow cytometry. **B** and **C**. Cells were pretreated with/without 10mM NAC for 1 h, and then treated with/without 8 μM LZ-205 for 24 h. The apoptosis were analyzed with flow cytometry. Values were Means ± SD for at least three independent experiments (*p<0.05 and ^*^*p<0.01 compared with Control; *^#^p*<0.05 and *^##^p*<0.01 compared with LZ-205 treated group).

The rise of the level of ROS can result in ER stress, mitochondrial dysfunction and ultimately apoptosis [7]. To assess the effects of LZ-205 on ROS induction, we determined the production of ROS using a carboxy-H_2_DCFDA probe, which can be converted to a green fluorescent product, carboxy-DCF, by oxidation. The results suggested that LZ-205 upregulated the level of ROS in NCI-H460 cells, as shown in Figure 4A. Additionally, the same phenomenon was detected in A549 and GLC82 cells (Supplementary Figure S1C). N-acetylcysteine (NAC), a ROS scavenger, was used to further examine whether ROS effected the induction of apoptosis by LZ-205. The results revealed that the level of ROS induced by LZ-205 was partly reduced by NAC (Figure 4A). In addition, Annexin V/PI staining showed that NAC partly inhibited the LZ-205-induced apoptosis (70.95±8.66% in the LZ-205 group vs. 22.19±8.62% in the NAC+LZ-205 group, Figure 4B and 4C). Thus, these results indicated that LZ-205 increased the level of ROS, which in turn could be partly responsible for the induction of apoptosis.

### Involvement of ER stress in LZ-205-induced apoptosis

**Figure 5:**
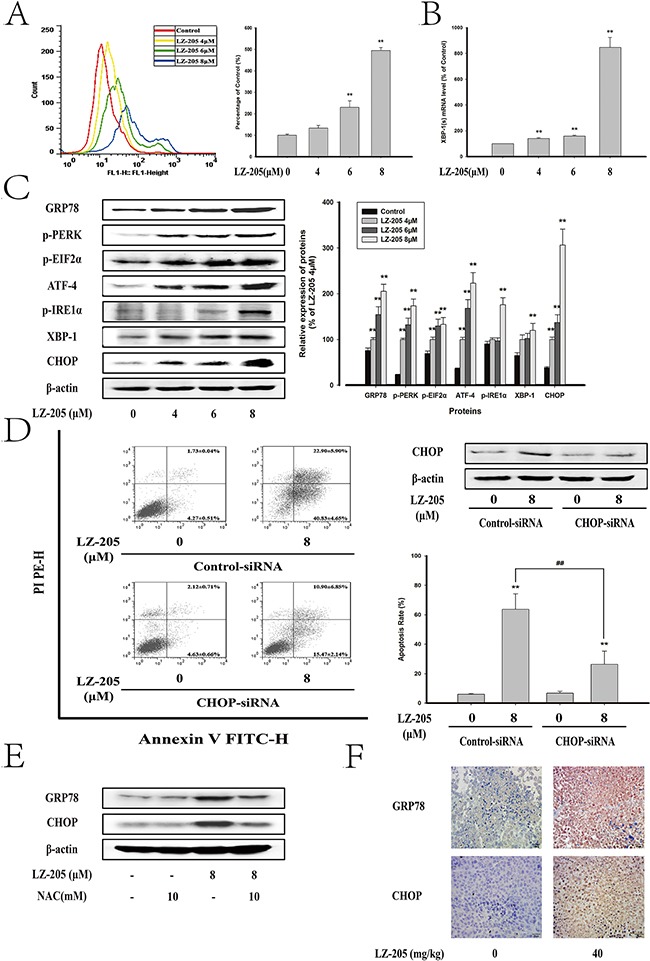
LZ-205 induced apoptosis by initiating ER Stress Cells were treated with 4, 6 and 8 μM LZ-205 for 24 h **A**. The Ga^2+^ level was detected by flow cytometry. **B**. LZ-205 induced alternative splicing of *XBP1* mRNA. It was analyzed by Quantitative real-time PCR. **C**. The protein of GRP78, p-PERK, p-EIF2α, ATF-4, p-IRE1α, XBP-1 and CHOP were analyzed by western blotting. **D**. Cells were transfected with control-siRNA or CHOP-siRNA and incubated for 8 h, and then treated with/without 8 μM LZ-205 for 24 h. The protein of CHOP was detected by western blotting. Then the apoptosis rate was detected by Annexin V/PI double staining. **E**. Cells were pretreated with/without 10 mM NAC for 1 h, and then treated with/without 8 μM LZ-205 for 24 h. The expression of GRP78 and CHOP were analyzed by western blotting. **F**. Immunohistochemical detection of GRP78 and CHOP protein levels in mouse transplantation tumor tissues (100×). Data were Means ± SD for three independent experiments (*p<0.05 and ^*^*p<0.01 compared with Control or Control-siRNA-transfected group; *^#^p*<0.05 and *^##^p*<0.01 compared with LZ-205 + Control-siRNA treated group).

The ER stress-induced apoptosis is always accompanied by Ca^2+^ overloading [9]. The level of Ca^2+^ is shown in Figure 5A and Supplementary Figure S1D. A concentration-dependent increase in Ca^2+^ level was observed in LZ-205 treated cells. To determine the impact of ER stress on LZ-205-induced apoptosis, western blot analysis of regarding ER stress pathway-related proteins expression was conducted. The results indicated that LZ-205 induced the protein expression levels of GRP78, p-PERK and p-EIF2α, ATF4, p-IRE1α, XBP-1 and CHOP (Figure 5C). We also found increased expression levels of GRP78 and CHOP in A549 and GLC82 cells (Supplementary Figure S1E). An active spliced form of XBP1 (XBP1s), that triggers a distinct set of UPR-induced genes, which participate in protein folding in the ER, represents a major event triggering UPR signaling [14]. Accordingly, we evaluated the splicing of *XBP1* by quantitative real-time PCR and found that spliced *XBP1* mRNA level was clearly increased by LZ-205, which indicated IRE1-XBP-1 pathway were activated (Figure 5B). Overall these data suggested that LZ-205 triggers ER stress.

To further examine the role of ER stress in LZ-205-induced apoptosis, LZ-205-treated NCI-H460 cells were transiently transfected with control-siRNA or CHOP-siRNA and analyzed for apoptosis. The data shown in Figure 5D demonstrated a successful transfection. Additionally, CHOP-siRNA pretreatment significantly suppressed the LZ-205-induced apoptosis (69.24±0.59% in the LZ-205+control-siRNA -treated group vs. 34.69±7.04% in the LZ-205+CHOP-siRNA-treated group). The results indicated that CHOP promoted LZ-205-induced apoptosis.

In addition, NAC was used to reduce the ROS level and then the ER stress was evaluated. The results showed that NAC inhibited the LZ-205 upregulated expression of GRP78 and CHOP as shown in Figure 5E, which made it clear that LZ-205-induced ROS trigger the ER stress. Furthermore, the tissue sample IHC results revealed higher expression of the protein of GRP78 and CHOP in the LZ-205-treated group (Figure 5F).

All the above data verified that LZ-205 triggered ER stress *in vivo and in vitro*.

### LZ-205 exhibited potential anti-tumor effect with low toxicity *in vivo*

**Figure 6:**
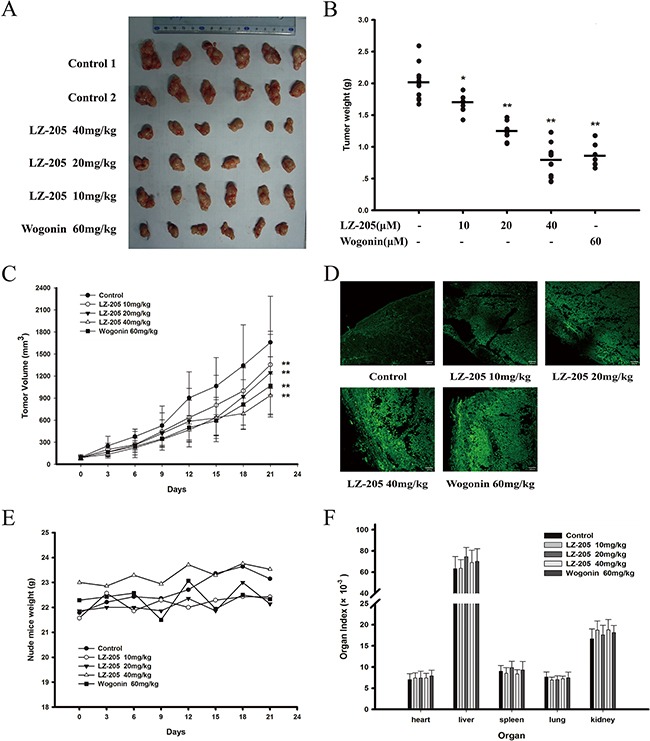
LZ-205 has a potential antitumor effect and low toxicity *in vivo* The transplanted mice NCI-H460 no-small cell lung cancers were treated with 10, 20 and 40 mg/kg of LZ-205 and 60mg/kg of wogonin by *i.v*. once every two days. Control group was treated with normal saline. **A**. Resected human non-small lung cancer tumor images from the experimental. **B**. The tumor weights were tested. **C**. Tumor volume results (100×) were given as Means ± SD. **D**. Detected the DNA damage with TUNEL assay. **E**. Nude mice weight was recorded once every three days. **F**. Main organs index of mice (organ weight/body weight) was used to evaluate the toxicity of LZ-205

A nude mouse model bearing inoculated H460 tumor was utilized to assess the antitumor effect of LZ-205 *in vivo*. The measured tumor volume further confirmed the significant reduction of the tumor size in the LZ-205 treatment group. Indeed, the 21-day treatment with LZ-205 (10, 20 and 40 mg/kg) or Wogonin (60 mg/kg) showed significant inhibitory effects on the growth of inoculated H460 tumors in mice (Figure 6A). Additionally, as shown in Figure 6B, LZ-205 inhibited the weight of the tumor, which was decreased by 61% in the group treated with 40mg/kg of LZ-205. A similar effect was observed with 60mg/kg Wogonin regimen, which showed an inhibitory rate of 58%. Treatment with LZ-205 at 10 mg/kg and 20 mg/kg resulted in 16% and 39% of tumor weight loss respectively. Compared with the control group, tumor volume in the LZ-205 (10, 20, and 40 mg/kg) and Wogonin (60 mg/kg) groups decreased by 18.31%, 24.87%, 43.52%, and 35.66%, respectively (Figure 6C). The TUNEL assay was used to analyze the apoptotic cells in the tumor tissues. Increased fluorescence signal indicated the extent of DNA damage induced by LZ-205 (Figure 6D). These results demonstrated that LZ-205 inhibited the growth of the transplanted tumors by inducing apoptosis in a dose-dependent manner.

Meanwhile, measurement and calculation of the mice weight and organ index revealed no significant change in any group (Figure 6E and 6F). In addition, hematological parameters results also indicated that there were no noticeable changes in the analyzed parameters in the tested animals and the standard ranges for the mice are listed in Table 1.

**Table 1:** Hematology profile in non-tumor bearing athymic nude mice administered with normal saline

Hematological parameters	Control	10mg/kg	20mg/kg	40mg/kg	standard
White blood cells (×10^3/μL)	4.97/5/7.59	6.07/5.25/6.64	3.56/5.54/N.D.	4.8/5.53/N.D.	4.5-9.1
Platelet (×10^3/μL)	716/978/652	1012/748/758	1082/349/1258	757/740/1011	115-1037
Band neutrophils(%)	0/0/0	0/0/0	0/0/0	0/0/0	0-1
Lymphocytes(%)	49.34/53.04/48.14	57.44/57/4/50.8	52.3/46.5/63.2	62.1/54.5/55	49-82
Eosinophils(%)	0.04/0.04/0.04	0.04/0.04/0.04	0.24/0.04/0.04	0.04/0.24/0.04	0-3
Monocytes	4.74/4.44/3.64	4.94/4.84/5.44	7.14/9.04/7.94	6.74/5.14/4.44	2-8
Mean corpuscular volume (fL)	51.6/49.4/49.3	50.9/47.5/52.4	47.5/51/50	49.7/47.2/51.1	41-60
Hematocrit (%)	43.9/46/43.1	44.2/43/48.9	50.2/49.2/42.9	47.9/46.8/44.1	34-50
Basophils (%)	0.04/0.24/0.04	0.04/0.04/0.04	0.04/0.04/0.44	0.04/0.24/0.4	0-3
Mean corpuscular hemoglobin (pg)	15.9/14.6/15.3	15.6/14.6/15.3	15.3/14.1/15.3	14.5/15.4/15.3	13-19
Mean corpuscular hemoglobin concentration (%)	30.8/29.6/31.1	30.5/30.7/29.2	30.1/31.1/30.3	30.5/30.1/30.6	30-39
Red blood cells (×10^3/μL)	8.5/9.32/8.75	8.68/9.05/9.33	9.9/10.82/8.52	10.08/9.17/8.82	7.51-9.66
Hemoglobin (g/dl)	13.5/13.6/13.4	13.5/13.2/14.3	15.1/15.3/13	14.6/14.1/13.5	12.8-16.1

Three mice per group were used. Standard ranges were obtained in house from 100 normal BALB/c mice of 8–12 weeks age.

In conclusion, LZ-205 inhibited the growth of H460 xenografts in nude mice in a dose-dependent manner without major side-effects. Additionally, the compound could dramatically induce apoptosis *in vivo*.

## DISCUSSION

Currently, more and more attention has been paid to flavonoids, like wogonin, as a novel source of anticancer drugs and new chemotherapy adjuvants to improve the efficacy of chemotherapeutic agents and to diminish their side effects. Wogonin induces apoptosis in hepatocellular carcinoma cells through the caspase 3 pathway and alternative expression of p21 protein [15–17] or by activating other pathways [18, 19]. In addition, many wogonin derivatives have been reported to possess potential anti-tumor properties, such as LYG-202, LW-214, etc. [20, 21]. As a new flavonoid compound similar in structure to Wogonin, LZ-205 induced lung cancer cell apoptosis in the present study. Actually, the apoptotic effect of 8 μM of LZ-205 treatment was stronger than that of 120 μM of Wogonin (66% vs. 33%). In the nude mouse model, LZ-205 exhibited the same growth inhibition ability as Wogonin. Although according to the National Comprehensive Cancer Network (NCCN) Guidelines for lung cancer, chemotherapy regimens, including platinum drugs, will prolong survival and improve symptom control, their use can be limited by granulocyte and platelet inhibition and renal toxicity. LZ-205 was found to have no significant influence on the hematological system and mice normal tissues, suggesting a potential low toxicity compared with traditional chemotherapeutic drugs. Moreover, LZ-205 is more water solubility than Wogonin, which makes it easier to be developed into a clinical drug.

ROS could be generated in diverse biological systems, and they also served as important determinants in cell signaling pathways involved in regulating proliferation, apoptosis and senescence. Above all, ROS is an important mediator of various chemotherapeutic agents [22]. Yu, J. Q. found that Wogonin altered ROS levels and induced cell death in human hepatoma cells [23]. In our study, LZ-205 increased the intracellular levels of ROS and induced apoptosis in human lung cancer cells, both of which were attenuated by the addition of NAC, a ROS scavenger. Oxidative stress is defined as a condition with an impaired prooxidant/antioxidant balance in a cell. Once cellular biomolecules confront severe oxidative damage, cell viability would be promoted [24]. ROS increase the level of oxidized proteins, which are identified as misfolded proteins in the ER and eventually trigger the UPR pathway. Meanwhile, LZ-205 increased the level of Ca^2+^, which is also implicated in the depletion of ER calcium. These findings indicate that ER stress could be involved in LZ-205-induced apoptosis. ER stress triggers apoptosis through two distinct mechanisms: one is the alteration in intracellular calcium levels and the other one is the accumulation of unfolded or misfolded proteins [25]. Actually, the PERK-EIF2α-ATF4 and IRE1α-XBP1 pathways were activated by LZ-205. CHOP is an important target gene driven by ATF4 and XBP1 and a proapoptotic transcription factor that reduces Bcl-2 and promotes the translocation of Bax [26, 27]. Silencing of the CHOP expression also reduced the LZ-205-triggered apoptosis induced by ER stress in lung cancer cells.

The intrinsic or mitochondrial apoptotic pathway plays a key role in accomplishing ER stress-induced apoptosis, and the collapse of the membrane potential (ΔΨm) is considered to be a hallmark of this pathway. Mitochondrial dysfunction induces the release of Cyt-c from the mitochondria, which triggers the caspase-dependent pathway and ultimately causes apoptosis [28]. AIF, another pro-apoptosis protein, translocates from the mitochondria to the nucleus, where it leads to DNA fragmentation and chromatin condensation [29]. Our study found that LZ-205 caused a decrease in the ΔΨm, couple with a release of Cyt-c and a nuclear translocation of AIF, which suggested that the intrinsic pathway takes part in the LZ-205 induced apoptosis. The proteins of the Bcl-2 family are key regulators of apoptosis and a slight imbalance of these proteins may lead to either inhibition or promotion of cell death. The ratio of Bax/Bcl-2 usually represents the extent of mitochondrial outer membrane permeabilization [30]. Accordingly, we investigated the Bcl-2 family of proteins to further evaluate the effect of LZ-205 on mitochondria. The results showed that LZ-205 increased the ratio of Bax/Bcl-2 and the expression of Bim and Noxa, which have been proven to induce Cyt-c release. Besides, a decrease of pro-caspase 9 and 3 suggested a caspase cascade was activated by LZ-205. Ultimately, we concluded that mitochondrial dysfunction contributed to LZ-205 induced apoptosis.

Noteworthy, neither NAC nor CHOP-siRNA can completely reverse the LZ-205-induced apoptosis. We speculated that there must be a pathway independent of ROS and ER stress involved in LZ-205-induced apoptosis. Previous studies found that Wogonin induces apoptosis through the Fas-related extrinsic signaling pathways in human osteosarcoma U-2 OS cells [18] and human leukemia CEM T cells [31, 32]. In the present study, LZ-205 also increased Fas and FasL expression and subsequently activated caspase 8. Moreover, pretreatment with a caspase 8 inhibitor significantly increased cell viability in the LZ-205-treated NCI-H460 cells. In addition, LZ-205 consistently increased truncated BID (tBID) levels which is normally the result of caspase 8 activation [33]. Thus, LZ-205 also triggers the extrinsic apoptotic pathway. Further research on the death receptor pathway including Fas, TNFα and Trail is in ongoing.

**Figure 7:**
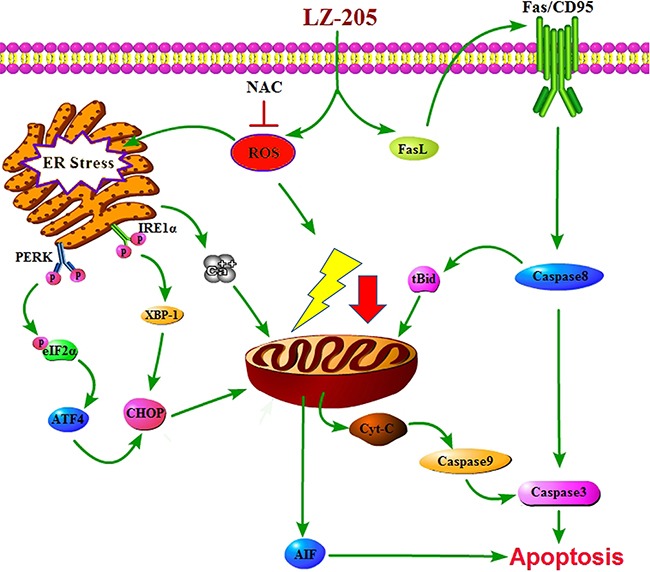
Schematic model for the mechanisms of LZ-205-induced apoptosis in human lung cancer cells LZ-205 induces apoptosis through multiple apoptotic pathways, including extrinsic apoptotic pathway and ROS-mediated-ER stress-dependent pathway.

In conclusion, our study revealed that LZ-205 inhibits the growth of lung cancer via the induction of apoptosis i*n vivo* and *in vitro*. LZ-205 triggered ROS-medicated ER stress thereby inducing apoptosis, as shown in Figure 7. Additionally, the extrinsic apoptotic pathway was found to be involved in this process. Our research provides a detailed explanation of the effect of LZ-205 on lung cancer cells and the molecular mechanism involved, and proposes this compound as a promising therapeutic agent against lung cancer in humans.

## MATERIALS AND METHODS

### Cell culture and reagents

The human non-small lung cancer NCI-H460 and A549 cell lines and human poorly differentiated lung adenocarcinoma GLC82 cell line were purchased from Cell Bank of Shanghai Institute of Biochemistry and & Cell Biology, Chinese Academy of Sciences. Cells were cultured in RPMI-1640 medium (Gibco, Thermo Fisher Scientific, USA) containing 10% fetal bovine serum (Gibco, Thermo Fisher Scientific, USA), 100 units/mL penicillin and 100 μg/mL streptomycin. Exponentially growing cultures were maintained in a humidified atmosphere of 5% CO_2_ at 37°C.

LZ-205 (purity 99.31%) was applied in sterile water to 0.01M and stored at -20°C. The concentrations used here were 4, 6 and 8 μM *in vitro*, and freshly diluted with sterile water to final con-centration. MTT [3-(4, 5-dimethylthiazol-2-yl)-2, 5-diphenytetrazoliumbromide] (Ronkonkoma, NY) was dissolved in 0.01 M PBS. Z-IETD-FMK and Z-VAD-FMK were obtained from Selleck (Shanghai, China) and was dissolved in DMSO to 5mM. N-acetyl-L-cysteine (NAC) was obtained from Sigma-Aldrich (USA) and dissolved in sterile water. Primary antibodies of ATF4, BID, Cyt-c, eIF2α, Noxa, PERK, p-PERK, p-eIF2α, Caspase 3, Caspase 8, Caspase 9, and β-actin were purchased from Santa Cruz Biotechnology (California, USA). Antibodies of AIF, Bim, and CHOP were purchased from Cell Signaling Technology (Beverly, MA, USA). Antibodies including Bax, Bcl-2, COX-IV, Fas, FasL, GRP78 and Histones were the purchased from Bioworld (USA). Annexin V-FITC Apoptosis Detection Kit was purchased from Vazyme (Nanjing, China).

### Cell viability assay

Cells were seeded into 96-well plates with 1×10^4^/well in 100 μL culture medium. LZ-205 were dissolved into sterile water and added into cells at different concentration. Cells were incubated with LZ-205 for 24 h before MTT assay [34].

### DAPI staining

Cells were cultured in 6-well tissue culture plates and then treated with the indicated concentration of drug in 24 h. At the end of incubation, the cells were fixed, washed with PBS and incubated with DAPI (1 μg/mL), which was purchased from Santa Cruz (USA). Apoptotic cells were determined by the staining of cell nucleus with the DAPI under fluorescence microscope (Olympus, Japan) with a peak excitation wavelength of 340 nm.

### Annexin V/PI staining

NCI-H460, A549 and GLC82 were seeded into 6-well plates and treated with LZ-205 or Wogonin. Cells were then harvested and washed with ice-cold PBS. According to the manufacturer's instructions, cells were stained with Annexin V and Propidium Iodide (PI) in binding buffer for 10 min. Samples were detected and analyzed by flow cytometry (FACSCalibur, BD, USA).

### Measurement of ROS formation [35]

NCI-H460, A549 and GLC82 were plated on 6-well plates, allowed to attach, and exposed to NAC for 1 h. Then cells were treated with LZ-205 for 12 h. Cells were harvested and incubated with 10 μM DCFH-DA (Beyotime Institute of Biotechnology, China) at 37°C for 30 min. After washed by serum-free, the fluorescence intensity was measured by flow cytometry.

### Detection of intracellular Ca^2+^ level

Cells were treated with LZ-205 for 24 h and then loaded with 1μM Fluo-3 AM (Beyotime, China) which combined with Ca^2+^ and produced strong fluorescence. After incubating for 45 min at 37°C in the dark, the cells were resuspended with PBS and the fluorescence intensity were measured by flow cytometry.

### Mitochondrial membrane potential (ΔΨm) assessment

Briefly, Cells were treated with LZ-205 for 24 h. Then cells were harvested, washed and incubated with JC-1((Beyotime Institute of Biotechnology, China). Samples were analyzed by flow cytometry (FACS Calibur, Becton Dickinson) with settings of FL1 (Green, FITC) at 530 nm and FL2 (Red, PE) at 590 nm, respectively.

### Subcellular fractionation [36]

NCI-H460 cells were treated with LZ-205 for 24 h and then collected. Mitochondrial and cytosolic fractions proteins were performed using cytosol/mitochondria fractionation kit (KeyGen Biotech, China) according to the following protocol.

### Quantitative real-time PCR

Total RNA isolation and real-time PCR were performed as previously described. The primers in the reaction were used as follows:

*Actin* (forward, 5’-GATCTGGCACCACACCT TCT-3’; reverse, 5’-GGGGTGTTGAAGGTCTCAAA-3’);

spliced (s) *xbp-1* (forward, 5’-GAGTCCGCAG CAGGTG-3’; reverse, 5’- GTGTCAGAGTCCATGG GA-3’).

### Transient transfection with CHOP small interfering RNA (siRNA)

NCI-H460 cells were grown to 60% confluence. Then either CHOP-siRNA (30 pmol/μL) or control-siRNA added into the cells with PepMute siRNA Transfection Reagent (SignaGen Laboratories, Rockville, MD). 8 hours later, the cells were harvested for further experiment.

### Western blot analysis [37]

Cells were lysed with a mixture of Pierce RIPA Buffer (Thermo, USA), and a debris was removed by centrifugation at 12,000 Xg for 20 min at 4°C. The concentration of total proteins was determined by using the BCA assay method (Thermo, Massachusetts, USA). After added by loading buffer and denaturation, protein samples (with 100 μg) were electrophoresed and transferred to nitrocellulose membranes. Blots were blocked for 2 h at room temperature, with 5% nonfat milk (BIO-RAD, USA) in PBS, and then incubated with primary antibodies for 1 h at 37°C and overnight at 4°C, which was followed by incubation with IRDye 800-labeled secondary antibodies (KPL, Gaithersburg, MD, USA) for 1 h at room temperature in the dark. Detection was performed by the Odyssey Infrared Imaging System (LI-COR, Lincoln, NE, USA).

### Animal model

Specific pathogen free (SPF) BALB/c nude mice (Shanghai Slac Laboratory Animal Co. Ltd. China) with body weight of 18-22 g and age of 35-40 days were raised in SPF Animal Laboratory of China Pharmaceutical University. All mice were subcutaneously inoculated with injections of 1×10^6^ cells. After 12-14 days, tumor sizes were determined using micrometer calipers, and then nude mice with similar tumor volume (neither too large nor too small) were randomly divided them into 5 groups (with 6 nude mice/group): saline control group, LZ-205 10, 20, 40 mg/kg group and wogonin 60 mg/kg group. All groups were administered intravenously every two days. Three weeks later, the nude mice were killed, and the tumor xenografts were removed and measured. Tumor volume (TV) was calculated using the following formula: TV (mm^3^) = D/2×d^2^, where D and d are the longest and the shortest diameters, respectively. All the animals were weighed every three days and monitored for mortality throughout the experimental period to assess toxicity of the treatments.

### Immunohistochemistry

The expression of GRP78, CHOP and FasL in tumor tissues of nude mice model was assessed to the method described previously [38], using a Goat-anti-mouse antibody and an Ultra-Sensitive TMSAP kit (Maixin-Bio Co, Fuzhou, China).

### Statistical analysis

All experiments are detected in triplicate (n=3) and expressed as mean ± SEM. We use the software of SigmaPlot, GraphPad and Excel to analyze these data and Student's *t*-test and two-way ANOVA to analyze the difference between sets of data.

## SUPPLEMENTARY FIGURE


