# Supplementary material for: Activation of endoplasmic reticulum stress and the extrinsic apoptotic pathway in human lung cancer cells by the new synthetic flavonoid, LZ-205
Source: Oncotarget. 2016 Nov 24;7(52):87257–70. doi: 10.18632/oncotarget.13535 (PMC5349986; doi:10.18632/oncotarget.13535)
Supplement: Supplementary file 1 [file oncotarget-07-87257-s001.pdf]

Activation of endoplasmic reticulum stress and the extrinsic apoptotic pathway in human lung cancer cells by the new synthetic flavonoid, LZ-205

SUPPLEMENTARY FIGURE

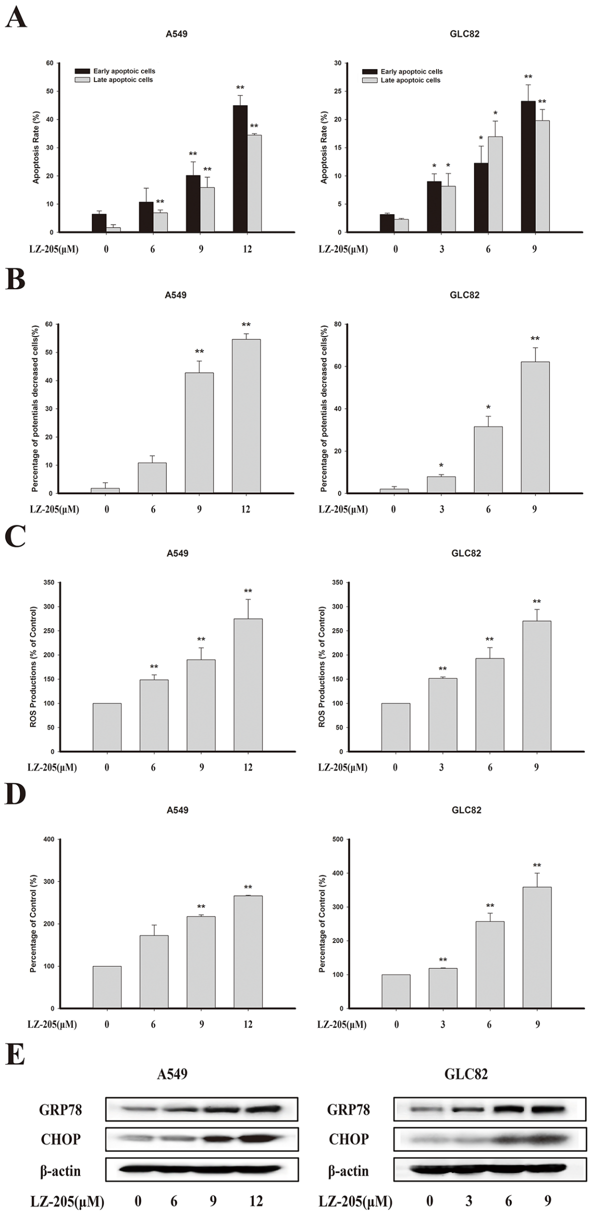

Supplementary Figure S1: LZ-205 induced A549 and GLC82 cells apoptosis by ROS-mediated ER stress. A459 cells were treated with 6, 9, and 12μM LZ-205 for 24h (12h for ROS assay), GLC82 cells were treated with 3, 6, and 9μM LZ-205 (12h for ROS assay). **A.** Annexin V/PI double-staining assay was analyzed by flow cytometry. **B.** Cells were stained with JC-1 and detected by flow cytometry. Then the percentage of ΔΨ collapsed cells was analyzed. **C.** ROS level was detected by flow cytometry. **D.** The Ga^{2+} level was detected by flow cytometry. **E.** The protein of PERK, p-PERK, GRP78, ATF4, eIF2α, p-eIF2α, and CHOP were analyzed by western blotting. Data were shown as Means ± SD for three independent experiments ($^{*}p$<0.05 and $^{**}p$<0.01 compared with control).
